# Does the “Tian-Ren-He-Yi” Belief System Promote Corporate Environmental Performance?

**DOI:** 10.3389/fpsyg.2022.886114

**Published:** 2022-04-18

**Authors:** Jieji Lai, Bin Liu, Hong Wang

**Affiliations:** ^1^School of Economics and Business Administration, Chongqing University, Chongqing, China; ^2^School of Accounting, Zhejiang Gongshang University, Zhejiang, China

**Keywords:** Tian-ren-he-yi, belief system, corporate environmental performance, corporate sustainable development, local pollution prevention

## Abstract

Buddhism and Taoism are among two of the major religions in China. Their “Tian-ren-he-yi” belief system promotes a conception of harmony between man and nature, which is an important guide for the construction of ecological civilization in China. Using data from Chinese listed manufacturing companies, this paper explores the impact and mechanism of Chinese local religious beliefs on corporate environmental governance. The results indicate that this belief system can improve corporate environmental performance. Furthermore, mechanism tests show that the “Tian-ren-he-yi” belief system promotes corporate environmental practices by reducing the pressure on management to achieve economic performance goals and increasing investment in environmental protection. Finally, the impact of the belief system on corporate environmental performance is more efficacious when the enterprises are under weak external supervision. Our results imply that the traditional religious culture is an important factor that influences corporate environmental performance in Chinese business practices.

## Introduction

The rapid development of global industry brings a significant increase in economic growth, but it also generates lots of environmental problems, such as air pollution and water pollution. In the early stage of a country’s development, pursuing economic goals at the expense of the environment seems inevitable. A working group led by the Asian Development Bank released a report (Towards an Environmentally Sustainable Future: A National Environmental Analysis of the People’s Republic of China) noting that a large number of motor vehicles and the rapid expansion of industry have all led to a serious environmental pollution problem in China. Environmental pollution harms not only the health and wellbeing of residents, but also the sustainable development of ecology. Fortunately, the Chinese government attached great importance to this problem and actively takes corresponding measures to protect the environment. Especially after the 18th National Congress of the Communist Party of China, which occurred in 2012, the following provisions were issued in clear text, such as improving processes related to the cadre-performance appraisal system, considering the environmental impact assessments of economic development, and incorporating ecological benefits into strategic planning. We can also see that to implement these measures, enterprises at all levels as participants must strive to meet the requirements of national economic development while emphasizing environmental protection. Corporate environmental performance has become an important indicator for evaluating ecological policy and environmental protection, and it is a critical component of constructing an ecological civilization world-wide.

The International Organization for Standardization (ISO) provides a useful tool to evaluate corporate environmental practices (e.g., ISO14031). It acknowledges differences in geographical, environmental, and technical conditions of various organizations and constructs an “environmental performance standard database” which subdivides environmental performance indicators into environmental status-indicators and environmental performance indicators. The corporate environmental performance evaluation system in China is based on it. According to our review, scholars hold a unanimous view that the relationship between corporate environmental performance and financial performance is positive. There is a temporary reduction in financial performance after a company fulfills its environmental obligations, but in the long run, environmental performance becomes a source of competitive advantage for corporations and improves their financial performance (Gallego-Alvarez et al., 2011; Paul et al., 2011; Trump and Guenther, 2017; Alexopoulos et al., 2018). Currently, scholars are increasingly focused on variables that affect corporate environmental performance, which are classified into internal and external factors. Internal factors relate to the existential characteristics of the firm such as its financial position and profitability (Angelia and Surya, 2015; Pavlos et al., 2019), and internal corporate governance factors (Williams, 2003; Deckop and Merriman, 2006; Bear et al., 2010; Barnard, 2011; Walls et al., 2012; Khan et al., 2021). External factors typically come from the government and market, such as external institutional pressure (Bradford and Fraser, 2008; Bye and Klemetsen, 2018; Ruiqian and Ramakrishnan, 2018), government regulations and subsidies (Karine and Kjetil, 2006; Christmann and Taylor, 2011; Cemoglu and Aghion, 2012; Hong et al., 2021), public opinion pressure (Yamaguchi, 2008), and the degree of competition in the product market (Zhu and Sarkis, 2007). These studies reveal factors that influence corporate environmental performance to a degree that is measurable and predictable, which implies that the environmental regulations play a role in restraining and regulating corporate actions and thus improving their environmental performance. However, researchers should note that informal systems such as religion and their cultural effects work in tandem with formal institutions to promote the progress of society. Especially considering China’s current transitional context of emphasizing economic development while protecting the environment, informal institutions such as religious culture occupy an increasingly important position. As analysts, it is not being sufficient to confine ourselves to accepting, assimilating, and improving formal systems while ignoring the informal systems that have slowly developed over thousands of years of history and have had a profound impact (Allen et al., 2005). Therefore, we consider the religious and cultural dimension to corporate activities and advocates that ecological concepts (such as an ecological moral consciousness in informal systems) can be an entry point to explore the intrinsic link between religious culture and corporate environmental performance. We expand the research on the drivers of corporate environmental performance and provide insights on how to win the battle against pollution while still promoting the construction of a prosperous ecological civilization in practice.

The empirical results demonstrate that (1) The “Tian-ren-he-yi” belief system can promote corporate environmental performance, compared with firms that do not operate on these principles. (2) The belief system promotes corporate environmental practices by reducing the pressure on management to achieve economic performance goals and increasing investment in environmental protection. (3) The belief system can serve as an informal, but beneficial, complement to existing institutions. (4) The impact of the belief system on corporate environmental performance is more efficacious when the enterprises are under weak external supervision.

Our study contributes to both theory and practice in several ways. First, we complement the literature on the influence of religion and its cultural effects on corporate business practices. Prior studies mainly focused on the relationship between religion and business ethics (Weaver and Agle, 2002; Conroy and Emerson, 2004), equity pricing (Ghoul et al., 2012), financial reporting (Dyreng et al., 2012; McGuire et al., 2012), earnings management (Callen et al., 2011), and corporate social responsibility (Zeng et al., 2016). Although some researchers have paid attention to the connection between religion, culture, and environment in the corporate sphere (Arbuckle and Konisky, 2015; Wei et al., 2017), they focus on corporate environmental investment, rather than corporate environmental performance. Our study addresses this gap and documents systematic evidence on how religious culture affects corporate environmental performance.

Second, by focusing on how the domains of cultural transmission and environmental protection intersect, we shed light on issues related to cultural and environmental protection. We also demonstrate that since religion plays an important role in human affairs, it can be used as a complementary schema to corporate governance mechanisms to regulate corporate behavior.

Third, our findings have policy implications. Punishment is not the only way to protect the environment: incorporating religious beliefs to foster and promote an environmentally friendly corporate culture is also an option.

## Theoretical Analysis and Research Hypothesis

### The Meaning of “Tian-Ren-He-Yi”

Chinese civilization originated from the practice of farming, which in its early days had primitive approaches and low productivity; in particular, the harvest of crops depended entirely on natural conditions such as geography and climate. Therefore, the relationship between humans and the natural environment was necessarily closer than it is in the modern era, and the early wise men were concerned with the concept of ecology nonetheless. After being developed gradually through Taoism and Buddhism, the “Tian-ren-he-yi” belief system was integrated into the daily life of Chinese people. The understanding and advocacy of this sensitive relationship between human beings and nature constitute ecological ethics in traditional Chinese religion and culture. The basic meaning of “Tian-ren-he-yi” is summarized as follows: First, human beings and other life-forms are a part of nature (i.e., all life in the natural system has its inherent value and has the right to live). Therefore, all forms of life are equal, and human beings cannot destroy the natural habitat without considering a moral limitation. Furthermore, moral principles are consistent with the laws of nature; therefore, human beings can properly and readily resolve the relationship between their interests and the interests and development of other life-forms in the world. There is no dilemma (or dichotomy) between ecology and ethics: human beings are able to develop their interests in harmony with nature, and they ought to do so. In a phrase, it means “the nature and mankind combined as one” or “nature-human harmony” (Peng et al., 2016).

### The “Tian-Ren-He-Yi” Belief System and Corporate Environmental Performance

Religious culture is the product of social norms that lead to the construction of informal systems. As such, religious culture has a measurable impact on human behavior, and it plays a macro-role in regulating society, economy, and life-processes (Williamson, 2000). Religion provides guidance and clarity for the goals and activities pursued by humankind, which shapes the mindset and behavior of the faithful (Ip, 2009). At the same time, the institutional attribute of religion fills in the behavior of humans with more specific details and regulations, thus forming an implicit and informal constraint on human behavior (and also exercising “soft power” invisibly but comprehensively; Du et al., 2015). We believe that the “Tian-ren-he-yi” belief system can influence the corporate environmental performance in the following ways:

First, the “Tian-ren-he-yi” belief system exerts the “soft power” that restrains and regulates human behavior.

The Taoist concept of “Tian-ren-he-yi” considers that man and nature are a unified “whole.” Pursuit of harmonious coexistence is not a matter of applying rational thinking, but rather a matter of the way, humans experience their existence. Furthermore, human beings benefit from a commitment to harmony and charity if they wish to maintain harmony between the whole of nature and its life-forms. Taoism promotes the practice of respecting and revering nature, thus inspiring people to respect and protect nature from over-exploitation. Taoism likewise believes that all life-forms are equal so human beings should respect other life-forms and make use of them with limitation, which is a typical embodiment of ecological thought and practice. Those who kills the goose that lays the golden eggs will sink themselves and others into a boundless desire for material enjoyment that will bring them nothing but danger. Taoism firmly advocates that people take action to protect nature: The Taoist classics warn their readers that burning mountains and polluting land lead to the destruction of vegetation. Furthermore, the classics also stipulate on how to protect land and water resources, such as not destroying mountains and rivers without restrictions; Taoism prohibits its followers from throwing any contaminants in the water. This is the fundamental idea of environmental protection.

The Buddhist concept of “Tian-ren-he-yi” holds that human beings and other life-forms are closely related, and that the individuals and their surroundings are complementary but inseparable. Furthermore, Buddhism strictly forbids its believers from harming others for their private interests. The Buddhist classics say as: “The earth has the same root of people, and all life-forms are on par with people,” which is a typical expression of ecological holism through Buddhism. Buddhist beliefs firmly hold to cause-and-effect transmigration, which means that if someone treats nature recklessly and abuses natural resources, then this person will reap the consequences of their actions. The doctrine of “Tian-ren-he-yi” encourages its followers to show mercy to sentient life and to not pollute the environment, instead, humans should do more to protect the natural ecology. Buddhism is mindful of self-restraint and the reduction of material needs on the part of its followers; it is excessive greed that has led to man’s unlimited desire to exploit nature.

Social norms that are formed by religion’s interaction with human culture can be considered factors that affect the behavior of individuals within each religious or cultural domain (Conroy and Emerson, 2004; Ghoul et al., 2012). As a part of religious culture, the belief system has deep impact on its believers. Such an eco-ethical notion can promote environmental values within corporations and increase the importance of environmental issues to decision makers. We believe that increased environmental awareness will motivate companies to propose protections that are beneficial to the environment and ultimately improve their environmental performance. Based on the “social identity theory,” the social norms originated and conducted by religions can lead to group-level common behaviors (Tajfel, 1986), that is to say, the norms in the groups greatly shape individual characteristics. McGuire et al. (2012) also argued that, regardless of whether the executives believe in religion, religion has influence on their attitudes, judgments, and decisions. Even if they were not religious believers in the enterprise, the “Tian-ren-he-yi” belief system can put external pressure on companies by fostering public advocacy campaigns and creating a regional cultural environment that is conducive to ecological thinking. Companies are bound to adopt environmental values to gain social acceptance, which affects their environmental performance.

Second, the “Tian-ren-he-yi” belief system exerts a “better vision,” which demonstrates the excitation power of religious culture.

Both Buddhism and Taoism describe an ideal notion for their believers to advocate. The ideal as described by Buddhism is that the follower will find nirvana and achieve paradise after the end of life: the paradise is orderly, beautiful, and peaceful. The ideal as described by Taoism is that the followers of Taoism enter an immortal world after practicing their discipline: a world in which there are no worries but only happiness and ecological balance. The religious culture encourages the believers to act conscientiously and work hard during their life in order to cultivate themselves to attain righteousness and enter the Elysian Fields. At the same time, the believers are encouraged to commit to the vision of “an earthly paradise” and build a better world through their efforts when they are in this life on Earth (Cooper and James, 2017). We believe that the concepts of environmental protection and restoration of damaged ecosystems (which are promoted by Buddhism and Taoism) will motivate companies to increase their environmental protection efforts and increase their investment in green technology. These actions ultimately promote the environmental performance of companies.

Based on the above exposition, we formulate the following hypothesis 1:

*H1*: *Ceteris paribus*, the “Tian-ren-he-yi” belief system promotes better corporate environmental performance.

## Research Design

### Variables

#### Corporate Environmental Performance

Corporate environmental performance (CEP) refers to the effects and accomplishments of business activities on account of environmental protection and controls of pollution. In the existing literature, the measurement of corporate environmental performance is done through a series of approaches called the evaluation-system method, the pollution emission method, the environmental capital expenditure method, and the eco-efficiency method. The latter three measurement approaches are comparatively limited and cannot provide a complete evaluation of corporate environmental performance, so this paper utilizes the evaluation-system method. Specifically, this paper comprehensively measures corporate environmental performance in the following eight aspects: (1) whether the company has developed or applied innovative products, equipment, or technologies that are beneficial to the environment. (2) Whether the company has policies, measures, or technologies to reduce emissions of waste gas, wastewater, waste residue, and greenhouse gases. (3) Whether the company uses renewable energy or adopts policies or measures for a circular economy. (4) Whether the company has energy-saving policies or technologies. (5) Whether the company has green office policies or measures. (6) Whether the company’s environmental management system has passed ISO 14001 certification. (7) Whether the company has received environmental recognition or other positive evaluation. (8) Whether the company has other advantages in environmental aspects that are not covered in the above indicators. If a condition is satisfied for the items above, the item is assigned a value of 1 or otherwise a value of 0. All scores are summed, and the higher the score, the better the CEP.

#### Measurement of Religious Culture (“Tian-Ren-He-Yi” Belief System)

There are several ways to measure religiosity according to the existing literature: first, the percentage of the religious population in the region (Dyreng et al., 2012); second, the number of religious places *per capita* (Ghoul et al., 2012); third, regional religious participation as based on questionnaires (McGuire et al., 2012); and fourth, the number of worship places around the company (Du, 2013). On the other hand, there is often a lack of open and uniform information on the characteristics, distribution, and religious participation of populations within administrative regions in China (Du, 2013). Therefore, we use the number of worship places (Buddhist and Taoist monasteries) in the same city as listed companies in order to quantify religiosity at the firm-level (i.e., as to overcome the lack of available statistics). Specifically, the locations of key Buddhist and Taoist temples were manually collected and matched with the registered locations of listed companies based on the “Report on the Identification of Key Buddhist and Taoist Temples in Han Areas Nationwide” issued by the People’s Republic of China on April 9, 1983. On this basis, a firm-level religious impact index was calculated.

Other control variables include Growth rate, Lnsize, return on assets (Roa), enterprise value (Tbq), tangible asset ratio (Tang), and other factors that affect corporate environmental performance. Specific variables were defined as shown in Table 1.

**Table 1:** Variable definitions.

Variables	Definitions
CEP	The natural logarithm of the total scores calculated based on the evaluation of the firm’s environmental protection behavior and effectiveness
REC	The natural logarithm of the number of Taoist and Buddhist temples in the city where the listed companies are registered
Lnsize	The natural logarithm of company’s total assets
Tbq	(Current market value + non-current market value + book value of liabilities)/book value of assets
Roa	The ratio of earnings before interest and tax (EBIT) to total assets
Lev	The ratio of total liabilities to total assets
Growth	(*t*–stage Total assets—*t*-1stage Total assets)/(*t*-1stage Total assets)
Tang	(net fixed assets + sum of inventories)/total assets
Top1	The shareholding ratio of the largest shareholder in year *t*
Esu	The natural logarithm of analysts who analyze the company in the current year
Reg	The score of urban pollution regulatory information disclosure index

### Data and Sample Selection

We used a research sample that includes all Chinese A-share listed manufacturing companies whose information was printed from 2008 to 2018. We used the following parameters to screen the sample: (1) we excluded companies from firm-year observations that have transaction statuses of special treatment (ST), suspension from trading (*ST), or particular transfer (PT). (2) We removed companies from firm-year observations if any variables in the main regression model are missing. The final sample consists of 6,346 firm-year observations. The financial data used in this paper were obtained from the CSMAR database, and the religious data were collected manually based on the Report on the Identification of National Key Buddhist and Taoist Temples in Han Areas that was issued by the People’s Republic of China on April 9, 1983. All of the continuous variables listed are winsorized at the 1% level.

### Empirical Models

To test hypothesis H1, we construct the following model by referring to Clarkson et al. (2008) and Walls et al. (2012):


(1) $$\begin{array}{l} {\mathrm{CEP}}_{i,t}=\alpha_{0}+\alpha_{1}REC_{i,t}+\alpha_{2}Lnsize_{i,t-1}+\\ \alpha_{3}Tbq_{i,t-1}+\alpha_{4}Roa_{i,t-1}+\alpha_{5}Lev_{i,t-1}+\\ \alpha_{6}Growth_{i,t-1}+\alpha_{7}Tang_{i,t-1}+\\ \alpha_{8}Top1_{i,t-1}+\alpha_{9}Esu_{i,t-1}\;+\\ \alpha_{10}Reg_{i,t-1}+\sum Ind+\sum Year+\varepsilon_{i,t} \end{array}$$

In the above model, the dependent variable CEP is a proxy variable for the level of corporate environmental performance, the independent variable REC is a proxy variable for the degree of religious and cultural influence. In this model, industry and year-effects are controlled and *ε* is the residual. The regression coefficient *α*_1_ indicates the degree of influence of religious culture on corporate environmental performance. If *α*_1_ is positive and statistically significant, it indicates that the “Tian-ren-he-yi” belief system promotes better corporate environmental performance.

## Empirical Result

### Descriptive Statistics

Table 2 reports the descriptive statistics for all variables that were used in this study. As shown in Table 2, the average of CEP is 1.292, which contains a minimum value of 0 and a maximum value of 2.079, thus indicating that corporate environmental performance can be improved. REC had an average value of 2.935, a minimum of 0, a standard deviation of 0.925, and a maximum of 4.771. The relatively large SD of REC indicates that there is a wide variation in the distribution of religious culture, which forms the basis for our study. The remaining variables are not further described.

**Table 2:** Sample selection and sample distribution.

Variables	Mean	*SD*	Min	Median	P75	Max
CEP	1.292	0.475	0	1.386	1.609	2.079
REC	2.935	0.925	1.091	3.045	3.638	4.771
Lnsize	23.043	1.462	13.786	22.875	23.932	26.229
Tbq	2.011	1.439	0.851	1.565	2.290	31.122
Roa	0.043	0.062	−0.618	0.037	0.068	0.429
Lev	0.492	0.200	0.101	0.504	0.640	0.996
Growth	0.179	0.387	−0.362	0.107	0.229	3.072
Tang	0.244	0.188	−0.206	0.202	0.362	0.621
Top1	0.392	9.913	0.124	0.375	0.442	1
Esu	2.328	0.791	0.693	2.397	2.891	4.159
Reg	3.940	0.315	2.398	4.011	4.174	4.396

### Pearson Correlation

Table 3 lists the Pearson correlation coefficients of major variables. The results show that the correlation coefficients between CEP and REC are positive such that they are statistically significant at the 5% level.

**Table 3:** Pearson correlation.

Variables	CEP	REC	Lnsize	Tbq	Roa	Lev	Growth	Tang	Top1
CEP	1.000								
REC	0.062^***^	1.000							
Lnsize	0.299^***^	−0.037^***^	1.000						
Tbq	−0.169^***^	0.006	−0.264^***^	1.000					
Roa	0.010	−0.008	0.031^***^	0.076^***^	1.000				
Lev	0.142^***^	−0.016^***^	0.223^***^	−0.125^***^	−0.385^***^	1.000			
Growth	−0.034^***^	−0.002	0.123^***^	0.004	0.188^***^	−0.021^***^	1.000		
Tang	0.127^**^	0.013^***^	0.098^***^	−0.010^**^	−0.053^***^	0.052^**^	−0.091^***^	1.000	
Top1	−0.030^**^	0.012^**^	0.122^***^	0.111^***^	0.056^***^	−0.019^***^	0.041^***^	−0.060^***^	1.000

**, *** represent significant at the 5%, and 1% significance level, respectively.

### Hypotheses Test

The previous hypothesis predicted that a religious culture that promotes “Tian-ren-he-yi” will improve corporate environmental performance. Table 4 reports the OLS regression results. To address a concern about potential serial correction problems associated with unbalanced panel data, we computed and reported all *t*- values using robust SEs adjusted for clustering at the firm-level.

**Table 4:** The linear model regression results.

Variables	CEP (1)	CEP (2)
REC	0.013^**^ (2.08)	0.018^***^ (2.85)
Lnsize		0.112^***^ (20.79)
Tbq		−0.005 (−1.17)
Roa		0.017^**^ (2.29)
Lev		−0.003 (−0.09)
Growth		−0.003^***^ (−4.31)
Tang		0.052 (1.18)
Top1		0.033 (0.84)
Esu		0.007 (0.87)
Reg		0.070^***^ (3.22)
_Cons	0.663^***^ (4.46)	−1.969^***^ (−9.69)
Year FE	Yes	Yes
Ind FE	Yes	Yes
N	6,346	6,346
Adj_R2	0.14	0.21

** *and* *** represent significant at the 5 and 1% significance level, respectively.

*t* values are provided in parentheses.

As shown in column (1), the coefficient of REC is positive such that it is statistically significant at the 5% level. When a series of relevant control variables are added, the regression coefficient of REC remains positive and statistically significant at the 1% confidence level (column 2). This indicates that religious culture improves corporate environmental performance and that the more Buddhist and Taoist monasteries in where the firm is registered, the better environmental performance there will be, verifying Hypothesis 1.

### Endogenous Discussion

During the Qing Dynasty, the Confucian classics became the official textbook for the imperial examinations, and the scholars naturally became the propagators of “Tian-ren-he-yi” belief system. In the Qing Dynasty, the printing and publication of books were basically carried out by the official printing bureaus. Due to the imbalance of the geographical distribution of the official printing bureaus and the inconvenient transportation, there were differences in the circulation of books in different regions, and thus, there were differences in the dissemination of the “Tian-ren-he-yi” belief system. After entering modern society, these printing bureaus were closed down due to the impact of advanced Western publishing technologies. Therefore, there is no direct relationship between these printing bureaus and the corporate environmental performance, satisfying` the conditions of correlation and exogeneity. So we add the official printing bureaus (OPB) of the Qing Dynasty as the instrumental variable. According to the test results in Table 5, there is a positive correlation between instrumental variables and explanatory variables, at the 1% confidence level, predictive explanatory variables and explained variables are also significantly and positively correlated, which indicates that religious culture can promote corporate environmental performance.

**Table 5:** Regression results of instrumental variables.

Models\Variables	1st stage	2nd stage
	REC	CEP
REC		0.066^***^ (5.21)
OPB	0.930^***^ (52.39)	
Lnsize	−0.034^***^ (−3.42)	0.115^***^ (21.16)
Tbq	−0.017^**^ (−2.23)	−0.003 (−0.75)
Roa	0.032^***^ (2.43)	0.013^*^ (1.90)
Lev	0.178^***^ (2.72)	−0.009 (−0.26)
Growth	0.004 (0.52)	−0.003^***^ (−4.07)
Tang	−0.015 (−0.20)	0.053 (1.22)
Top1	−0.002^***^ (−3.19)	0.056 (1.42)
Esu	0.074^***^ (5.58)	0.004 (0.06)
Reg	−1.152^***^ (−28.19)	0.110^***^ (4.62)
_Cons	7.243^***^ (22.44)	−2.283^***^ (−10.30)
Year FE	Yes	Yes
Ind FE	Yes	Yes
*N*	6,346	6,346
Adj_R2	0.35	0.28
Shea’s partial R2	0.16	
*F*		623.26

*t* values are provided in parentheses.

* represents significant at the 10% significance level.

** represents significant at the 5% significance level.

*** represents significant at the 1% significance level.

### Robustness Test

The model was regressed by staggering control variables at stages to exclude control variables from playing a determinant role in the regression results. To further ensure the reliability of the main findings and to avoid conclusions formed by pure chance, we performed the following robustness tests.

#### Constructing the Differential Model (DID)

The impact of religious culture on corporate environmental performance can be inflected by capturing the changes in the environmental performance after a firm’s registration place migrates from an area of low religiosity to an area of high religiosity. Since the migration of firms’ registration place occurs in multi-batch and multi-year instances, this paper will utilize the dummy variable Migration so as to highlight the migrated companies. Specifically, regions are divided into two groups that are based on the level of religiosity: if a firm’s place of incorporation has migrated from a location of low religiosity to a location of high religiosity previously or in the current year, its dummy variable Migration is then marked with a value of 1, otherwise its Migration is marked 0. According to the premise of the difference-in-differences (DID) method, the dummy variable Migration can effectively capture the net-change in corporate environmental performance after the place of registration changes from a location with low religiosity to a location with high religiosity. The regression results of the DID model are reported in Table 6. As shown in column (1), the regression coefficient of Migration is positive and significant at the 5% confidence level. This further demonstrates that religious culture contributes to the improvement of corporate environmental performance.

**Table 6:** Robustness test results.

Models\Variables	DID	Tobit
	CEP (1)	CEP (2)
REC		0.096^***^ (4.21)
Migration	0.222^**^ (1.98)	
Lnsize	0.087^***^ (17.67)	0.362^***^ (19.74)
Tbq	−0.015^***^ (−3.29)	−0.056^***^ (−3.87)
Roa	0.033^***^ (4.34)	0.141^***^ (4.86)
Lev	−0.027 (−0.79)	−0.193 (−1.64)
Growth	−0.003^***^ (−2.99)	−0.008^*^ (−1.79)
Tang	0.252^***^ (7.87)	0.734^***^ (6.48)
Top1	−0.001 (−0.46)	−0.001 (−0.90)
Esu	0.030^***^ (3.90)	0.108^***^ (4.14)
Reg	0.029 (1.37)	0.227^***^ (3.43)
_Cons	−1.263^***^ (−6.97)	−6.48^***^ (−14.30)
Year FE	Yes	
Ind FE	Yes	
Firm FE	Yes	
N	6,346	6,346
Adj_R2/Pseudo R2	0.13	0.05

*t* values/*z* values are provided in parentheses.

* represents significant at the 10% significance level.

** represents significant at the 5% significance level.

*** represents significant at the 1% significance level.

#### Replacing the Regression Method (Tobit)

Since some of the explanatory variables in the sample have zero values, we use the Tobit regression model to retest the effect of religious culture on corporate environmental performance. The results are reported in Table 6. As shown in column (2), we find that the regression coefficient of REC is still positive such as to be statistically significant after replacing the regression method. The conclusion that religious culture improves corporate environmental performance is still valid.

## Further Analysis

### Possible Mechanisms

We have demonstrated the positive effect of religious culture on corporate environmental performance. In this section, we discuss the underlying mechanisms by which religion improves corporate environmental performance.

The rising global competition demands corporates to be more economically competitive. Meanwhile, the natural environment seems to deteriorate around the world and protecting the environment demands more attention. Obviously, both economic performance and environmental performance are important (Porter and Kramer, 2011). The dilemma is as: How do firms reconcile these different performance dimensions? If the corporations have only embraced an economically oriented strategy, in their pursuit for economic performance, they end up significantly polluting the environment. In existing studies, scholars have confirmed that corporate executives will overinvest in their companies under the pressure of meeting economic performance indicators (Kuusela and Maula, 2016). The long-term consequence of overinvestment is to ensure competition for natural resources and bring pollution and destruction to the environment. The “Tian-ren-he-yi” belief system insists that believers ought to understand the laws of nature and master moral limits when using natural resources. It avoids the polarization of dangerous extremes, such as too much emphasis on human needs or too little emphasis on the environment. This “ecological ethical connotation” will inevitably guide the behavior of believers and alleviate the utilitarian pursuit of economic benefits by the shareholders of companies (i.e., at the expense of the environment), thus relieving management of pressure to meet environmental performance indicators. At the same time, the “Tian-ren-he-yi” belief system shapes a corporate culture that promotes environmental protection and reduces the disagreement and friction among stakeholder parties in protecting the environment. In this way, corporations will respond more positively to ecological protection and make decisions that are friendly to the environment, such as by increasing investment in environmental protection in order to improve their environmental performance.

To verify the mechanisms, we follow Ma et al. (2019) to classify firms into two groups according to management economic performance pressure and regress them separately. A firm is defined as being in the group with high pressure if the firm’s average price-to-earnings ratio predicted by analysts is greater than the predicted annual industry median. The regression results are listed in Table 7. As shown in columns (1) and (2), the regression coefficient of REC is significantly positive only in the group with high pressure. It indicates that the impact of the belief system on corporate environmental performance is partly channeled by alleviating the pressure of management performance. Moreover, we also classify firms into two groups according to corporate environmental investment and regress them separately. The regression results are listed in Table 7. As shown in columns (3) and (4), the regression coefficient of REC is significantly positive only in the group with less corporate environmental investment. It indicates that the impact of the belief system on corporate environmental performance is partly channeled by increasing corporate environmental investment. To sum up, the “ecological ethics” contained within the traditional Chinese religions of Buddhism and Taoism alleviates the pressure of performance on the part of executives to reduce degradation to the environment through external regulation. At the same time, ecological ethics also help to increase the firms’ environmental investment in order to repair the natural environment (i.e., through internal regulation). The two channels work together to improve the corporate environmental performance.

**Table 7:** Mechanism test results.

	Management performance pressure		Corporate environmental investment	
Variables	High	Low	Less	More
	CEP (1)	CEP (2)	CEP (3)	CEP (4)
REC	0.021^**^ (2.47)	0.014 (1.52)	0.016^*^ (1.94)	0.019 (1.32)
Lnsize	0.111^***^ (15.70)	0.112^***^ (13.13)	0.107^***^ (14.83)	0.118^***^ (13.96)
Tbq	−0.003 (−0.57)	−0.003 (−0.45)	−0.003 (−0.41)	−0.006 (−1.02)
Roa	0.194 (1.20)	0.014 (1.11)	0.013 (1.21)	0.154 (0.82)
Lev	0.027 (0.57)	−0.018 (−0.32)	−0.002 (−0.05)	0.008 (0.16)
Growth	−0.003^***^ (−5.48)	−0.009 (−1.42)	−0.002^***^ (−4.17)	−0.007 (−1.63)
Tang	0.113^*^ (1.93)	−0.010 (−0.15)	−0.021 (−0.38)	0.156^**^ (2.20)
Top1	0.001 (0.45)	0.000 (0.77)	0.001 (1.09)	−0.001 (−1.10)
Esu	0.009 (0.86)	0.002 (0.15)	0.010 (0.96)	0.006 (0.49)
Reg	0.100^***^ (3.37)	0.035 (1.11)	0.051^*^ (1.87)	0.100^***^ (2.77)
_Cons	−2.102^***^ (−7.07)	−1.759^***^ (−6.17)	−1.817^***^ (−7.85)	−2.089^***^ (−5.39)
Year FE	Yes	Yes	Yes	Yes
Ind FE	Yes	Yes	Yes	Yes
N	3,680	2,666	3,776	2,570
Adj_R2	0.22	0.21	0.22	0.27

*t* values are provided in parentheses.

* represents significant at the 10% significance level.

** represents significant at the 5% significance level.

*** represents significant at the 1% significance level.

### The “Tian-Ren-He-Yi” Belief System and Corporate Environmental Performance: An Analysis Based on the Intensity of Regulations and External Supervision

Williamson (2000) documents four levels of “Social Analysis,” in which religion lies in the first and embedded level, the governance structures with transactions in third. Generally speaking, religion complements market governance mechanism. The introduction of external supervision can act as a restraint on management behavior through reputation maintenance and pressure mechanisms. When the intensity of external supervision increases, when a company has environmental problems, they can be easily detected and quickly transmitted to the outside world, thus damaging the company’s image and social reputation. To save corporations reputations, executives are bound to pay attention to environmental protection and make moves to improve corporate environmental performance. That is, external monitoring can weaken the role of religious culture in improving corporate environmental performance. Du et al. (2015) also show that religiosity can curtail unethical behaviors of managers, while this relationship is attenuated for firms with better external monitoring mechanisms. As an important information intermediary in the capital market, analysts play an important role in external supervision, and analysts’ attention to companies is an important supervisory force. Therefore, this paper classifies firms into two groups according to analysts’ concerns as being subject to strong and weak external monitoring, and we regress them separately. A company is defined as being in the group with stronger external oversight if the number of analysts it follows is greater than the median industry-annual number of analysts who follow it. The regression results are listed in Table 8. As shown in columns (1) and (2), the regression coefficient of REC is significantly positive only in the group with weak external oversight. It indicates that the impact of the belief system on corporate environmental performance is more efficacious when the enterprises are under weak external supervision.

**Table 8:** Heterogeneity analysis results.

	External supervision		Environmental regulation	
Variables	Weakly	Strongly	Weakly	Strongly
	CEP (1)	CEP (2)	CEP (3)	CEP (4)
REC	0.020^**^ (2.10)	0.014 (1.64)	0.027^***^ (2.93)	0.014 (1.50)
Lnsize	0.099^***^ (10.29)	0.115^***^ (15.99)	0.118^***^ (15.72)	0.106^***^ (13.34)
Tbq	−0.001 (−0.11)	−0.011^*^ (−1.66)	−0.002 (−0.36)	0.001 (0.14)
Roa	0.008 (0.90)	0.159 (1.06)	0.012 (1.15)	0.303^*^ (1.67)
Lev	−0.038 (−0.64)	0.062 (1.37)	0.038 (0.82)	−0.014 (−0.26)
Growth	−0.003^***^ (−4.12)	−0.011 (−1.21)	−0.003^***^ (−4.57)	−0.004 (−0.99)
Tang	0.080 (1.15)	0.012 (0.20)	0.034 (0.50)	0.053 (0.90)
Top1	0.001(0.52)	0.001 (1.09)	−0.001 (−0.62)	0.001 (1.52)
Esu			0.006 (0.58)	−0.002 (−0.18)
Reg	0.098^***^ (2.88)	0.047^*^ (1.66)		
_Cons	−1.698^***^ (−5.78)	−2.032^***^ (−7.22)	−1.625^***^ (−6.94)	−1.388^***^ (−7.48)
Year FE	Yes	Yes	Yes	Yes
Ind FE	Yes	Yes	Yes	Yes
N	2,995	3,351	3,249	3,097
Adj_R2	0.17	0.26	0.26	0.18

*t* values are provided in parentheses.

* represents significant at the 10% significance level.

** represents significant at the 5% significance level.

*** represents significant at the 1% significance level.

Both formal and informal systems are fundamental parts of the disciplinary approach in management, which plays an important role in the behavior and decision making of senior staff. Their decisions may be complementary or alternative to the goal of environmental preservation. In reality, the reasons for why companies improve their environmental performance are complex. They may choose to follow their ethical values or implement policies passively under the pressure of the institutional environment or because of social norms. In areas with strong environmental regulations, the formal legal system will force executives to take effective actions to improve corporate environmental performance. In this case, the role of religious culture in improving corporate environmental performance may be weakened. Therefore, we complete a further analysis to investigate the differences between the two types of regulatory environments. The Urban Pollution Regulatory Information Disclosure Index (PITI) is jointly developed by the Institute of Public and Environmental Affairs (IPE) and the Natural Resources Defense Council (NRDC). The index can effectively reflect the institutional constraints represented by the central government’s environmental surveillance, including its supervision by the Ministry of Ecology and Environment. We divided the firms into strongly and weakly regulated groups and conducted regression analyses on each group. The regression results are listed in Table 8. As shown in columns (3) and (4), the regression coefficient of REC is positive and statistically significant only in areas with weak regulation, which indicates that the effect of religious culture on improving corporate environmental performance is stronger in areas with weak regulation. The conclusion from this observation implies that religious culture serves as an important and implicit alternative governance mechanism that compensates for formal institutions when they are inadequate.

### The “Tian-Ren-He-Yi” Belief System and Corporate Environmental Performance: An Analysis Based on CEO’s Heterogeneity Characteristics

The upper echelon theory shows that age, educational background, and other characteristics effectively reflect people’s values and cognitive basis, and these values and cognitive basis will further affect people’s decision-making behavior (Hambrick and Mason, 1984). The spread and penetration of religious culture are a slow process, and it takes time to influence believers. So as CEOs get older, religious culture influences them more. The rich life experiences of older CEOs enable them to have a deeper understanding of “Tian-ren-he-yi” belief system, which makes them more willing to invest in corporate environmental governance. Being well-educated means that CEOs generally have a more complete body of knowledge that can shape better values and guide them to fulfill their corporate social responsibility in a more active way (Slater and Dixon-Fowler, 2010). And environmental protection is part of corporate social responsibility. Therefore, we believe that well-educated CEOs will spontaneously improve corporate environmental performance. That is, the educational background will weaken the role of religious culture in improving corporate environmental performance. We complete a further analysis to investigate how the different ages and education work in the relationship between religious culture and corporate environmental performance. We divided the firms into old age, Young age, Poor-educated, and well-educated four groups, and conducted regression analyses on each group. The regression results are listed in Table 9. As shown in columns (1) and (2), the regression coefficient of REC is positive and statistically significant only in the group with old ages, which indicates that the effect of religious culture on improving corporate environmental performance is stronger when the CEO with old age. As shown in columns (3) and (4), the regression coefficient of REC is significantly positive only in the group with poor-educated. It indicates that the educational background will weaken the role of religious culture in improving corporate environmental performance.

**Table 9:** Heterogeneity analysis results of corporate executives.

	Age of CEO		Educational background of CEO	
Variables	Old	Young	Poor-educated	Well-educated
	CEP (1)	CEP (2)	CEP (3)	CEP (4)
REC	0.031^***^ (3.31)	0.011 (1.26)	0.032^***^ (3.56)	0.001 (0.16)
Lnsize	0.118^***^ (15.24)	0.101^***^ (12.26)	0.130^***^ (16.42)	0.094^***^ (11.53)
Tbq	0.001 (0.17)	−0.007 (−1.21)	−0.005 (−0.61)	−0.007 (−1.33)
Roa	0.007 (0.54)	0.338^**^ (2.12)	0.018 (1.39)	0.515^***^ (3.34)
Lev	−0.025 (−0.45)	0.020 (0.41)	0.067 (1.36)	−0.013 (−0.23)
Growth	−0.002^***^ (−4.19)	−0.003 (−0.69)	−0.002^***^ (−3.78)	−0.011^*^ (−1.66)
Tang	0.041 (0.68)	0.071 (1.07)	0.008 (0.11)	0.162^***^ (2.75)
Top1	0.001 (0.89)	0.001 (0.71)	0.001^***^ (2.61)	−0.001^*^ (−1.76)
Esu	−0.004 (−0.36)	0.015 (1.35)	−0.011 (−0.98)	0.021^*^ (1.92)
Reg	0.086^***^ (2.85)	0.033 (1.03)	0.074^**^ (2.34)	0.064^**^ (2.16)
_Cons	−2.092^***^ (−7.99)	−2.032^***^ (−7.22)	−2.442^***^ (−8.73)	−1.352^***^ (−4.08)
Year FE	Yes	Yes	Yes	Yes
Ind FE	Yes	Yes	Yes	Yes
N	3,145	3,201	3,163	3,183
Adj_R2	0.23	0.22	0.24	0.22

*t* values are provided in parentheses.

* represents significant at the 10% significance level.

** represents significant at the 5% significance level.

*** represents significant at the 1% significance level.

## Summary and Conclusion

This paper explores the effects and mechanisms of religious culture (namely: “Tian-ren-he-yi” in Buddhism and Taoism) on corporate environmental performance in China. The empirical results indicate that the “Tian-ren-he-yi” belief system can improve corporate environmental performance. Furthermore, the “Tian-ren-he-yi” belief system promotes corporate environmental practices by reducing the pressure of management on achieving economic performance goals and increasing investment in environmental protection. We also find that the belief system can serve as an informal, but beneficial, complement to existing institutions. Finally, the impact of belief system on corporate environmental performance is more efficacious when the enterprises are under weak external supervision. By focusing on how the domains of cultural transmission and environmental protection intersect, we expand the research on the drivers of corporate environmental performance and provide insights on how to win the battle against pollution while still promoting the construction of a prosperous ecological civilization in practice. We also demonstrate that religion plays an important role in human affairs.

The findings of this paper suggest that the traditional religious culture of China improves the corporate environmental performance in Chinese business practices. Therefore, scholars and researchers should evaluate religious culture more comprehensively, rationally, and objectively when they are promoting sensible environmental policy that is specific to a region or nation. As an effective supplement to formal organizations and institutions, soft “restraining” forces such as religious culture plays an important governance and regulatory role; this indicates that it is feasible to draw wisdom from a traditional religious culture in order to solve environmental problems in contemporary times. While actively creating a legal environment that supports sensible environmental planning, the government should also consider the role of supervision that traditional religious culture plays in environmental performance. It is necessary for corporations to actively organize environmental activities and transform a rich ecological theory into a part of lived-corporate culture. Human beings ought to take multiple measures—and to make the necessary efforts—to create a comfortable living space for ourselves and our offspring.

## Data Availability Statement

Publicly available datasets were analyzed in this study. This data can be found at: https://cn.gtadata.com/.

## Author Contributions

BL and HW contributed to conception and design of the study and reviewed and revised the draft critically for important intellectual content. JL organized the database, performed the statistical analysis, and wrote the first draft of the manuscript. All authors contributed to the article and approved the submitted version.

## Funding

This research is supported by the Natural Science Foundation of Zhejiang Province (LQ20G020006).

## Conflict of Interest

The authors declare that the research was conducted in the absence of any commercial or financial relationships that could be construed as a potential conflict of interest.

## Publisher’s Note

All claims expressed in this article are solely those of the authors and do not necessarily represent those of their affiliated organizations, or those of the publisher, the editors and the reviewers. Any product that may be evaluated in this article, or claim that may be made by its manufacturer, is not guaranteed or endorsed by the publisher.
